# Environmental, Social, Governance Risk and Corporate Sustainable Growth Nexus: Quantile Regression Approach

**DOI:** 10.3390/ijerph182010865

**Published:** 2021-10-15

**Authors:** Xiaodong Teng, Yanzhi Wang, Aiguo Wang, Bao-Guang Chang, Kun-Shan Wu

**Affiliations:** 1 School of Accountancy, Shandong University of Finance and Economics, Jinan 250014, China; 20019411@sdufe.edu.cn (X.T.); 202208177@mail.sdufe.edu.cn (Y.W.); 19871490@sdufe.edu.cn (A.W.); 2 Department of Accounting, Tamkang University, New Taipei City 251301, Taiwan; 3 Department of Business Administration, Tamkang University, New Taipei City 251301, Taiwan

**Keywords:** ESG, corporate sustainable growth, sustainable growth rate, quantile regression

## Abstract

Despite a huge body of literature revealing that the effect of environmental, social and governance (ESG) scores on a firms’ financial performance and value, it lacks the empirical research on the nexus between corporate sustainable growth and ESG risk in the existing research. The paper aims to examine the nexus between ESG risk and corporate sustainable growth. This study utilizes a quantile regression approach to explore how ESG risk affects corporate sustainable growth (proxied by sustainable growth rate, SGR). The ordinary least squares estimation results confirm that ESG significantly negatively affects corporate sustainable growth. The quantile regression results reveal ESG risk has a significant negative effect on corporate sustainable growth in the upper quantiles of SGR, but not in the lower and median quantiles. The results show that the impact of ESG risk on the corporate sustainable growth is asymmetric and affected by the distribution of SGR. Furthermore, the research results identify that the negative relationship between ESG risk and corporate sustainable growth is particularly apparent for firms in environmentally sensitive industries. This study greatly contributes to existing literature, as with this detailed knowledge, managers can make decisions based on these associations and identify the most lucrative course of action.

## 1. Introduction

Sustainable growth is essential to a firm’s survival in the current competitive business market. Sustainable growth refers to the revenue growth that a company can achieve within its financial and operational constraints [1]. As such, it is a crucial embedded factor associated with a company’s survival and expansion [2,3,4,5,6,7,8,9]. Higgins’ [10] original sustainable growth rate (SGR) is defined as the maximum rate at which firm sales can increase without the depletion of financial resources. SGR is a financial indicator that many companies use to address potential growth issues, and companies can also use it to ensure long-term success [11]. Using SGR, investors and managers can begin to assess whether a company’s plans for future growth are realistic [12], as it provides useful information to ascertain the reasons for a firm’s success or failure [12,13]. Consequently, SGR provides an overall view of performance that grants investors and managers insight into the factors affecting growth rate, and how these relate to the income sources which maintain the company’s survival long-term [14]. Furthermore, facing the global sustainable development agenda, climate change mitigation and a shift toward to low-carbon economies [15], increasingly many companies require effective risk management. Companies should not only focus on traditional financial risks but also consider the environment, social and governance (ESG) risks [16,17].

ESG is regarded as a major factor in measuring the impact of corporate sustainability [18]. The ESG score (or ESG rating) refers to the extent to which enterprises are exposed to ESG-related issues, where ESG risk is the unmanaged portion of that exposure [19]. Company stakeholders are increasingly interested in ESG risk and the strategies for managing it [20]. Thus, this study’s main research question is whether ESG risk has an effect on SGR and if so, what are the specific effects of ESG risk on SGR?

The linkage between ESG scores and firm financial performance has been theoretically and empirically explored with inconclusive or contradictory results. Two conflicting theories seek to explain the effect of sustainability on firms’ financial performance: value development (e.g., stakeholder theory, agency theory) and value destruction (trade-off theory) [21,22,23]. The approach to value-creation theory suggests that costs are minimized, competitive advantage is reinforced, reputation and legitimacy is developed, performance is maximized, and corporate risk is minimized with ESG disclosure [21,22,24]. On the other side, the value destruction hypothesis implies that ESG disclosure activity is costly and detrimental for shareholders [21,22]. In addition, many studies have shown that net profit margin, asset turnover, dividend payment, financial leverage, non-interest income [12,13,14], intangible assets [25,26], working capital policy [27,28], environmental management [29] and other operating and financial performance indicators are related to SGR. Although the relationship between operating, financial performance and SGR has been extensively studied, the fact SGR can be influenced by non-financial components, such as ESG risk factors, cannot be ignored. The ESG risk rating is a concept developed by Sustainalytics in mid-2019 to measure a company’s exposure to ESG-related risks, replacing the ESG score previously used [22,30]. This shift in the perspective of the ESG score—from an indicator of sustainability virtue to an indicator of internal vulnerability—can highlight whether investors should treat the ESG label as an indicator of market performance [15].

Previous studies [31,32] show that the value creation effect of the ESG score lies in an industry’s characteristics. The effect is different particularly for companies in environmentally sensitive industries (for instance, chemical, gas, mining, and oil industries) that are polluting sectors and perceived as having high environmental risk; however, most of the surveyed prior research does not explore the influence of these industries.

Motivated by the abovementioned studies, this research aims to use the quantile regression (QR) method to test the effect of ESG risk (unmanaged ESG factors) on sustainable growth for different SGR quantiles, and further examines the relationship between ESG risk and SGR, and whether it differs between environmentally sensitive and non-sensitive industries. In addition, the traditional ordinary least squares (OLS) method focuses on the mean as a measure of location and can be inconsistent because it does not report the tails’ information of the data distribution [33]. Consequently, OLS regression results can be distorted. Moreover, the QR method is more robust and generates more efficient estimates than OLS regression because the QR method allows us to estimate the nexus between the dependent variable and its explanatory variables at any specific quantile.

This study contributes to the literature on ESG risk and SGR in several ways. First, the research attempts to fill the gap in prior studies by investigating the impact of ESG risk management on sustainable growth, and further understanding whether the ESGRISK-SGR relationship differs between environmentally sensitive and non-sensitive industries. Secondly, using QR method, this paper not only addresses the tail information of SGR, but also demonstrates the influence of ESG risk on different SGR quantiles. The test results contribute to ESG risk and SGR literatures by discovering new information concerning the association between ESG risk and SGR, specifically the varying influence of ESG risk on SGR in different quantiles.

The remainder of this article is structured as follows: Section 2 discusses the theoretical background, reviews earlier studies and hypotheses development; Section 3 discusses the research methodology and its econometric modelling and estimation approaches; Section 4 discusses data, a summary of the statistics, empirical results and robustness checking; and finally, in Section 5, the discussion and conclusions are presented.

## 2. Literature Reviews and Hypothesis Development

### 2.1. Sustainable Growth Rate

A firm’s sustainability can be assessed by its growth prospects. SGR refers to “the growth in revenues a firm can achieve given its current operating performance and financial constraints” [34]. Higgins [10] defines SGR as “the maximum rate at which a company’s sales can increase without depleting financial resources”. SGR is a firm’s long-term target growth rate [1] and has been used in some studies as a measure of sustainable growth [1,2,13,28].

SGR can help managers in financial planning [35], especially in terms of control and performance [28]. By understanding SGR, companies can plan their growth targets based on their ability to finance, avoiding the impact of financial difficulties and negative cash flows [36] that can lead to bankruptcy [37]. SGR can be used to measure the extent to which a business effectively uses its assets to generate profits as a source of internal financing to enable the business to continue to grow [28]. SGR is essential to a firm’s survival when faced with issues such as accessing resources, routine development, increasing legitimacy and sustaining boundaries.

The concept of SGR has been used by many researchers in previous studies. Arora et al. [13] used panel data regression to study the associations among the asset turnover, financial leverage, net profit margin, and SGR of the Indian manufacturing sector’s firms listed on the National Stock Exchange of India Ltd., between 1998 and 2014. Yu and Tsai [38] used the regression model to explore the effect of environmental policy on sustainable development in Chinese enterprises. Mamilla [12] used the linear regression method to study the relationship between asset efficiency, financial leverage, firm size liquidity, and SGR for the oil refineries industry in India. Al-Slehat and Altameemi [14] used the simple linear regression model to explore the relationship between non-interest revenue and SGR for commercial banks in Jordan. Ionita and Dinu [25] used the OLS model to assess the associations among intangible assets, SGR and firm value in firms listed on the Bucharest Stock Exchange in Romania. Ocak and Fındık [26] used OLS and Heckman’s two-stage estimation procedure to examine the impact of intangible assets on SGR and enterprise value in Turkey. Xu et al. [29] applied the two-stage least squares regression to explore the impacts of debt financing and environmental management on SGR in the Chinese tourism industry. Kuo and Chang [39] also applied the two stages least squares regression to investigate the effect of circular economy information on corporate economic sustainability in China. Sunardi et al. [28] investigated the association among conservative working capital policy, profitability, and SGR in the Indonesian manufacturing industry between 2013 and 2018 by using the panel data regression of stochastic effects estimation model. Chen et al. [2] studied the association between COVID-19, customer concentration and sustainable growth by using hierarchical linear model based on data from Chinese listed companies.

These researchers demonstrate the practical implications and the importance of SGR to different kinds of research; however, they only consider financial information, not non-financial factors.

### 2.2. ESG Risks

Sustainability risks refer to “the environmental, social or governance events or conditions, which, if they occur, have or may potentially have significant negative impacts on the assets, financial and earnings situation, or reputation of a supervised entity” [40]. Sustainability risks, or ESG risks, have gained attention among financial investors, stakeholders, and scholars [15,18,20,22,30,41,42,43], and an increasing number of investors rely on ESG rating providers to measure these risks.

Stakeholder theory highlights enterprise should create returns for stakeholders from ESG investments [44,45,46]. As a result, businesses will reap sustainable benefits and profits by focusing on the larger society through ESG [47]. Based on the theory of legality, a firm’s disclosure of ESG enables its commitment to society to be fulfilled and generates a sustainable profit pool. Companies and stakeholders expect these investments to yield long-term benefits [47]. Nevertheless, some researchers argue that these investments may not yield direct economic benefit [48,49]

As interest in ESG interest has grown, the number of ratings and metrics for non-financial information generated by sustainability rating agencies has been increasing annually [50]. While ESG metrics are important, they are highly subjective due to a lack of transparency [51], a lack of homogeneity in measurement [52], and lack of standardization [53]. Consequently, the results are inconsistent, particularly when the methodology is not disclosed [50,54,55,56].

Since mid-2019, ESG rating agencies have been producing ESG risk (un-managed share of ESG exposure) scores to replace the ESG scores previously used to measure companies’ ESG-related exposures [30]. As the new score replaces the old one as a baseline for sustainability, a high ESG score should now indicate lower risk from ESG factors [30]. To date, the Morningstar Portfolio Historical Sustainability Risk Score (Morningstar’s rating) is a historical holdings-based calculation using company-level ESG RISK Ratings from Sustainalytics [57].

### 2.3. SGR and ESG Risk Relationship

Despite a huge body of literature examining the effect of ESG scores on firms’ financial performance and value, there are inconsistent reports (positive, negative, neutral, and multiple relationships) and the study of the relationship between SGR and ESG risk is rare.

The poor management of ESG risks may damage the company’s credibility and reputation in the market and may negatively affect the sustainable growth of the company [22]. Khovrak [18] focused on comparing the ability of insurance companies to use ESG-driven methods to manage their sustainable development. The results show that the Sustainalytics’ ESG risk rating is the most appropriate rating to evaluate the ability of insurance companies to manage their sustainable development. Oprean-Stan et al. [22] applied multiple regression to investigate the associations among inadequate management of ESG factors, sustainability reporting, enterprise performance, and SGR for 50 companies in the STOXX Europe 50 Index. The results revealed that ESG risk had an insignificant effect on SGR; however, return on assets had a significant effect on SGR. Ferriani and Natoli [30] estimated the effect of the Sustainalytics’ ESG risk rating on investment fund flows during the outbreak of COVID-19. Their results show that low ESG risk positively affected inflows into equity funds during the COVID-19 crisis. Magnér [58] suggested that the number of sustainability indices may cause an obstacle for sustainable development rather than improved sustainability. Consequently, it is reasonable to expect ESG risk to impact SGR. Thus, the following hypothesis is proposed:

**Hypothesis** **1.** *ESG risk could negatively influence SGR. In other words, firms with higher ESG risk will have poor sustainable growth.*

ESG risks significantly vary across industries, for example, due to their environmental impact, a financial company and a mining company encounter different ESG risks [59]. Thus, to gain more insight into the ESG risk and SGR relationship, we also examined the possible impact of industry-specific characteristics. The authors [32,60] examined the relationship between ESG and financial performance in emerging markets between 2010 and 2015 and found that the value-creation effect of ESG in environmentally sensitive industries was lower than that of enterprises in non-sensitive industries. Consequently, it is reasonable to expect the ESG risk and SGR nexus differs between environmentally sensitive and non-sensitive industries. The second hypothesis is:

**Hypothesis** **2.** *The effect of ESG risk on SGR is greater for companies operating in environmentally sensitive industries than those in non-sensitive industries.*

## 3. Methodology

### 3.1. Data Source and Samples

This empirical research uses data from listed firms in Taiwan and was sourced from the Taiwan Economic Journal database (https://www.finasia.biz, accessed on 2 August 2021). TEJ database company is well designed and is currently one of the most accurate financial databases in Taiwan. It includes listed company information, stock market information, financial information, macroeconomics and commodities, Asian databases, etc. In addition, in 2020, the Taiwan Depositary and Clearing Corporation (TDCC), with reference to the Sustainalytics’ ESG RISK Ratings [19], began to disclosure TSE listed firms’ ESG practices on a voluntary basis. Thus, the corporate ESG risk ratings are taken from the TDCC ESG Risk Ratings database. Given the availability of the information (ESG risk rating scores) and the aim of performing analysis that reflects the current situation as far as possible, 2020 was selected as the research year. Thus, all 323 firms (excluding banking industry) with ESG practices in 2020 were selected as research subjects.

### 3.2. Variables

Dependent variable: SGR is used as a dependent variable to calculate the ROE (return on equity) multiplied RR (retention rate) [13,39]. RR is calculated as: RR = one firm’s dividend payout ratio. In brief, SGR is the growth rate after excluding the effect of the dividend payout rate from ROE.

Independent variable: The independent variable is a firm’s ESG Risk Rating (ESGRISK). Sustainalytics defines ESG risk as “the degree to which a company’s economic value is at risk driven by ESG factors or, more technically speaking, the magnitude of a company’s unmanaged ESG risks”. To measure ESGRISK, Morningstar’s rating, as well as the Environmental, Social, and Governance Portfolio Risk Score was used. In this study, the ESG risk scores of TSE listed firms were sourced from the TDCC, with reference to the Sustainalytics’ ESG RISK Ratings. Like the ESG scores, Morningstar’s rating uses a 0–100 scale, where lower scores are superior. ESG risk is divided by five risk levels: negligible (0–10), low (10–20), medium (20–30), high (30–40), and severe (40–100) [19].

Control variables: The control variables are company size (SIZE), financial leverage (LEV), firm age (AGE), net profit margin (NPM), and growth rate of owner’s equity (OEG), as previous studies found they determined SGR (or ROE) [39,61,62,63]. SIZE is measured by the natural logarithm of total assets; LEV = total liabilities/shareholder’s equity; AGE stands for firm age; NPM refers as the ratio of net income to sales; and OEG = (owner’s equity of current period-owner’s equity of previous period)/owner’s equity of previous period.

### 3.3. Research Model and Methods

OLS regression is often used to test hypothesis in sustainability research, as it captures the relationships at the mean. Nevertheless, focusing on central influences may lead to underestimation or overestimation of correlation coefficients, or failure to identify important associations. This may lead to misstatements and omitting information at the tail of the distribution [33]. Moreover, the QR method permits for a full range of conditional quantile functions, is more robust, and generates more efficient estimates than OLS regression because the QR method allows us to estimate the nexus between the dependent variable and its explanatory variables at any specific quantile. Besides, a well-known of quantile regression was introduced in Koenker and Bassett [64], based on conditional quantile functions. Quantile regression estimates the conditional median or the conditional quartile of the dependent variables for the given independent variables. This study uses the QR model suggested by Koenker and Bassett [64]:

$$Q_{\theta }\left(SGRq_{i}|X_{i}\right)=\beta_{0\theta }+\beta_{1\theta }ESGRISK_{i}+\beta_{2\theta }SIZE_{i}+\beta_{3\theta }LEV_{i}+\beta_{4\theta }AGE_{i}+\beta_{5\theta }NPM_{i}+\beta_{6\theta }OEG_{i}+\varepsilon_{\theta i}$$

where $Q_{\theta }\left(SGRq_{i}|X_{i}\right)$ is the θ-th quantile regression function. $SGRq_{i}$ is the SGR of *i* firm; $SIZE_{i}$ is measured as the natural logarithm of total assets of *i* firm; $LEV_{i}$ is the financial leverage of *i* firm; $AGE_{i}$ is the firm age of *i* firm; $NPM_{i}$ is measured as the ratio of net income to sales of *i* firm; $OEG_{i}$ is the growth rate of owner’s equity of *i* firm; and $\varepsilon_{\theta it}$ represents error terms at the θ-th quantile. This QR model examines the relationship between ESGRISK and SGR.

## 4. Results

### 4.1. Descriptive Statistic

**Figure 1:**
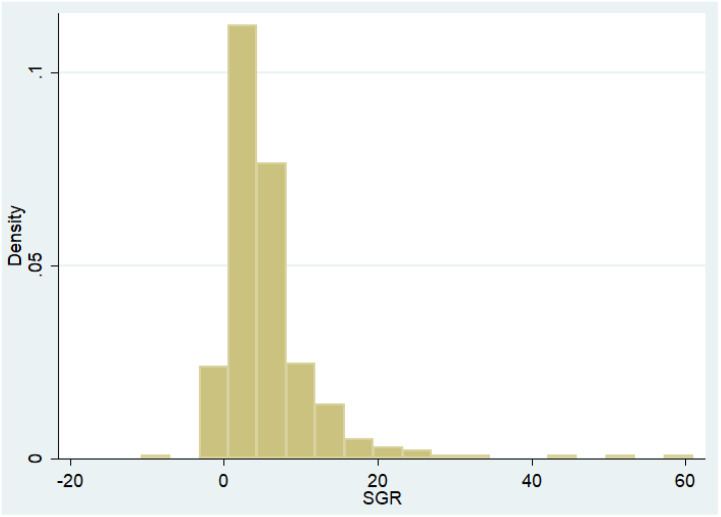
Histogram of SGR.

Table 1 presents a summary of statistics for all variables in the model. For 2020, the average level of ESGRISK for TSE listed firms with ESG practices is 26.76. Recalling the previously introduced corresponding grades of ESG RISK Ratings, the average overall ESGRISK score approximately corresponds to the medium risk scope [19]. For SGR, the mean value is 5.649, median 4.13, minimum −10.733, and maximum 61.035. The skewness and kurtosis moments indicate that the SGR of all TSE listed firms practicing ESG is distributed to the right and has fat tails. The normality test on SGR reports its Jacque–Bera statistic (=9012, *p*-value < 0.001), rejecting the null hypothesis of SGR as normally distributed. The SGR histogram (Figure 1) illustrates the non-normal distribution and skewed and heavily right-tailed distribution.

To test the collinearity of the independent variables, the variance inflation factors (VIF) were calculated. Hair et al. [65] states that a VIF equal to or lesser than 0.2 and equal to or greater than5 indicates the presence of multi-collinearity. Table 2 documents that the VIF values for all independent variables are less than two, confirming there is no serious collinearity problem [65].

### 4.2. Effect of ESGRISK on SGR

This section examines the effect of ESGRISK on SGR. Table 3 shows the results of the OLS and QR analysis for the full sample. Estimated coefficients are described for the 10th, 25th, 50th, 75th, and 90th quantiles of the conditional distribution of SGR. The OLS estimation reveals that the coefficient of ESGRISK has a significant negative impact on SGR. Regarding SGR, the QR results reveal the coefficient of ESGRISK is negative and significant in the upper quantiles (75th and 90th), which supports Hypothesis 1 (Table 3). It implies the ESGRISK influences firms with better performance and does not influence firms with poorer performance.

Among the control variables, OLS analysis shows that SIZE significantly negatively effects SGR, while QR analysis reveals that SIZE’s negative effect is predominantly in the upper quantiles. The OLS analysis of LEV confirms there is a significant positive effect; however, the QR analysis reveals this is predominantly in the 25th quantile. AGE is neither significant in the OLS model or the QR model. QR analysis reveals NPM has a positive effect in the 10th, 75th and 90th quantiles; however, this is not apparent in the OLS model’s results. Both OLS and QR show that OEG significantly positively affects SGR.

After excluding the financial holding firms, the OLS estimation also reveals that ESGRISK has a significant negative impact on SGR for non-financial holding firms. The QR estimation results suggest that the ESG significantly negatively affects SGR in the 90th quantile for non-financial holding firms.

### 4.3. Inter-Quantile Difference

The results specify the effect of ESGRISK on SGR is heterogeneous across the distributions of SGR. To test whether these differences are statistically significant, inter-quantile regressions are utilized to check for slope equality throughout the quantiles [64]. The F-test and corresponding *p*-value examines the uniformity of the coefficient between the upper and lower quantiles, using 200 replications with the bootstrap method (Table 4).

Table 4 presents the inter-quantile regression results of SGR. The parameter estimates of ESGRISK for Q(90/10) symmetrical quantiles were statistically different. For the explanatory variable ESGRISK, there is a statistically significant negative difference, indicating that ESGRISK has a significantly stronger negative influence on SGR in the 90th quantile than in the 10th quantile (Table 3).

**Figure 2:**
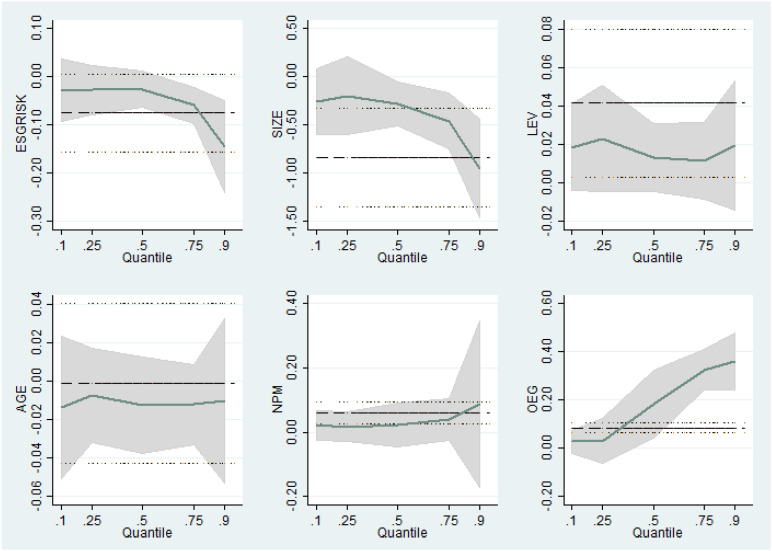
Estimated coefficients of OLS and QR, and their respective 95% confidence intervals.

Graphical evidence is used to depict how the independent variables affect the dependent variable. Figure 2 presents how each covariate’s effect varies across quantiles, and how they contrast with the OLS estimates for each explanatory variable. OLS and QR estimates are depicted using their respective 95% confidence intervals.

ESGRISK crosses the upper and lower bounds of significance level, which clearly shows the heterogeneous behavior of its relationship with the dependent variable, SGR. For the control variables, LEV, AGE and NPM lie in between the upper and lower bounds, whereas SIZE and OEG cross the significance level after a certain level of percentile, especially for the two highest (lowest) quantiles. This confirms the QR estimates are significantly different from the OLS estimates.

### 4.4. Effect of ESGRISK on SGR: Environmentally Sensitive vs. Non-Sensitive Industries

To test whether the ESGRISK-SGR relationship between environmentally sensitive and non-sensitive industries is different, we divide the whole company sample into environmentally sensitive and non-environmentally sensitive industries. The business activities of environmentally sensitive industry firms (such as oil, gas, paper, metal manufacturing, and chemical) have a direct impact on ESG issues [66,67,68,69] and are often perceived as polluting sectors that pose higher environmental risks [31,32].

There are 42 environmentally sensitive firms and 281 non-sensitive firms. Table 5 displays the OLS and QR estimation results for the environmentally sensitive and non-environmentally sensitive industries. For the environmental sensitive firms, the OLS estimation reveals that the ESGRISK has a significant negative impact on SGR, whilst the QR results reveal the ESGRISK is negative and significant in the lowest (10th) and highest (90th) quantiles, implying the ESGRISK influences firms with best or worst performance. However, ESGRISK has an insignificant effect on SGR for non-sensitive firms both OLS and QR method. Thus, Hypothesis 2 is supported. In summary, the initial findings on the significance of ESGRISK is confirmed in the environmentally sensitive firm subsample. This study yielded some interesting results by looking at the quantile regression tests.

### 4.5. Robustness Check

To check the robustness of the findings, the model was estimated using different subsamples. This article uses firm size as a proxy for information asymmetry, according to current literature convention. It is assumed that large companies have a low degree of information asymmetry and small companies have a high degree of information asymmetry and are treated as a separate sample [61]. The large firm subsample comprises of 150 firms with more than the average value of the natural logarithm of total assets, and the small firm subsample consists of 173 firms with fewer than the average value of the natural logarithm of total assets. Table 6 shows the estimation results for these subsamples. The results suggest ESGRISK has a significant negative impact on SGR for the large firm subsample, but an insignificant effect for the small firm subsample. Initial findings on the significance of ESGRISK is confirmed in the large firm subsample.

## 5. Discussion

This article makes a novel contribution to the ESG risk and SGR literatures by proposing that ESG risk has a negative effect on SGR. The empirical investigation of the negative effect of ESG risk on SGR of TSE listed firms uses both OLS and QR regression analysis. The results reflect and complement the relationship between ESG risk and SGR with several findings.

(1) OLS and QR estimation results indicate that ESG risk all had a significant negative effect on SGR. This finding agrees with the propositions of [30], who suggests that sustainability indices may represent obstacles for sustainable development rather than facilitators of improved sustainability. However, this result is inconsistent with Oprean-Stan et al. [22] and could be due to the different sample firms. Oprean-Stan et al.’s [22] sample firms are from developed countries (France and Germany) located in the European market, whereas this study’s sample is comprised of Taiwanese firms. As sustainability awareness is increasingly reflected in European legislation, ESG disclosures are no longer voluntary for firms, whereas they are still voluntary in Taiwan.
(2) The QR test results reveal the link between ESG risk and corporate sustainable growth is statistically significant in the upper quantiles of SGR. In other words, ESGRISK influences firms with better performance but does not influence firms with poorer performance.
(3) The negative relationship between ESG risk and SGR is particularly observed for environmentally sensitive firms. The result is similar to those reported by [32,60,66,70], however, this result is inconsistent with Ruan and Liu [71] and could be due to the different sample of firms. Ruan and Liu [71] reported the ESG rating had a significant negative correlation with firm performance in the non-environmentally sensitive industries.
(4) For environmentally sensitive firms, the QR test results reveal the link between ESG risk and corporate sustainable growth is statistically significant in the 10th and 90th quantiles of SGR. In other words, ESGRISK influences environmentally sensitive firms with worst or best performance.
(5) Stakeholder theory argues that low ESG risk rating improves the communication and trust of stakeholders towards enterprises, thus improving the sustainable growth of enterprises. In addition, consistent with the theory of legitimacy, ESG activities can be regarded as the intention of enterprises to pursue moral legitimacy in the social contract, and that such behavior will obtain higher enterprise value.

## 6. Conclusions

This study applied the QR method to investigate the impact of ESG risk on SGR for TSE listed firms with ESG disclosures. The empirical conclusions are as follows.

(1) ESGRISK has a significant negative impact on SGR as assessed by the OLS method. The QR method reveal that ESGRISK significantly negatively on SGR in the upper quantiles (75th and 90th).
(2) ESGRISK has a significant negative impact on SGR for environmentally sensitive industries, whereas has an insignificant effect on non-sensitive firms.

Based on the above conclusions, the empirical findings highlight two important implications:

(1) In terms of policymakers, using the ESG risk of Taiwanese firms can improve corporate sustainability practices, in line with sustainable growth planning, particularly for firms operating in environmentally sensitive industries.
(2) Since sustainability awareness is not legislated in Taiwan, all ESG practices are voluntary. Nevertheless, as per the theory of legitimacy, ESG disclosure activities can be regarded as the intention of enterprises to pursue the moral legitimacy, which is conducive to obtaining higher corporate value through sustainable enterprise development.

This research had several limitations. As sustainability awareness is not legislated in Taiwan and all ESG practices are voluntary, our data was from only the 323 listed firms whose ESG risk scores are available on the TDCC. Thus, more significant results could have been obtained if the sample size had been larger. Second, this research only studied data from Taiwan’s listed firms. Given that industries vary by country, it would be beneficial to confirm if data from other countries also found a significant negative ESGRISK–SGR relationship, and whether different ESGRISK influences SGR between countries, sectors and/or industries.

## Figures and Tables

**Table 1:** Descriptive statistics.

Variables	SGR	ESGRISK	SIZE	LEV	AGE	NPM	OEG
Mean	5.649	26.76	17.551	49.007	35.295	−1749.755	12.219
St. deviation	6.632	8.143	1.643	21.196	15.92	34,940.76	31.024
Min	−10.733	8.55	13.229	4.65	3	−701,412	−45.76
Max	61.035	48.19	23.117	96.85	75	681.47	446.26
10th percentile	0.653	17.05	15.829	22.11	15	1.23	−2.74
25th percentile	2.003	20.78	16.370	32.97	23	4.45	1.61
50th percentile	4.138	25.2	17.236	46.97	33	9.8	6.38
75th percentile	6.915	32.52	18.340	62.27	47	17.98	13.37
90th percentile	12.549	38.46	19.874	78.64	58	27.49	25.35
Skewness	3.78	0.313	0.933	0.31	0.388	−19.998	8.149
Kurtosis	25.695	2.451	3.782	2.504	2.372	400.938	101.11
Sample sizes	323	323	323	323	323	323	323

Note: SGR = sustainable growth rate; ESGRISK = ESG Risk Ratings; SIZE = company size; LEV = financial leverage; AGE = firm age; NPM = net profit margin; OEG = growth rate of owner’s equity.

**Table 2:** Bivariate correlation matrix.

Variables	(1)	(2)	(3)	(4)	(5)	(6)	(7)	VIF
(1) SGR	1							
(2) ESGRISK	−0.013	1						1.09
(3) SIZE	−0.110 *	−0.259 *	1					1.71
(4) LEV	−0.040	−0.168 *	0.613 *	1				1.63
(5) AGE	−0.065	0.019	0.160 *	0.075	1			1.04
(6) NPM	0.220 *	−0.055	0.070	0.022	0.087	1		1.04
(7) OEG	0.390 *	0.020	−0.106 *	−0.158 *	−0.124 *	0.040	1	1.05

Note: SGR = sustainable growth rate; ESGRISK = ESG Risk Ratings; SIZE = company size; LEV = financial leverage; AGE = firm age; NPM = net profit margin; OEG = growth rate of owner’s equity; * denotes statistical significance at 10%.

**Table 3:** Regression test results: ESGRISK on SGR.

		Full Sample				
Variables	OLS	Lower Quantiles		Median	Upper Quantiles	
		10th	25th	50th	75th	90th
ESGRISK	−0.0747 **	−0.0279	−0.0273	−0.0258	−0.0586 *	−0.1448 **
	(0.0325)	(0.0266)	(0.0268)	(0.0405)	(0.0323)	(0.0674)
SIZE	−0.8360 ***	−0.2578	−0.1945	−0.2744	−0.4559 **	−0.9498 **
	(0.3103)	(0.1700)	(0.1708)	(0.2583)	(0.2063)	(0.4302)
LEV	0.0416 **	0.0184	0.0232*	0.0132	0.0116	0.0196
	(0.0206)	(0.0128)	(0.0129)	(0.0194)	(0.0155)	(0.0324)
AGE	−0.0011	−0.0138	−0.0075	−0.0125	−0.0121	−0.0104
	(0.0169)	(0.0137)	(0.0138)	(0.0208)	(0.0166)	(0.0346)
NPM	0.0610	0.0218 *	0.0173	0.0213	0.0409 ***	0.0861 ***
	(0.0430)	(0.0113)	(0.0113)	(0.0171)	(0.0137)	(0.0286)
OEG	0.0855 *	0.0272 ***	0.0302 ***	0.1835 ***	0.3267 ***	0.3602 ***
	(0.0495)	(0.0073)	(0.0074)	(0.0112)	(0.0089)	(0.0186)
Constant	18.3695 ***	5.1789 *	4.7680	7.9158 *	12.7914 ***	24.8764 ***
	(4.9638)	(2.9311)	(2.9452)	(4.4548)	(3.5579)	(7.4188)
Sample size	323	323	323	323	323	323
Adj R-square/Pseudo R^2^	0.2169	0.0796	0.0760	0.1351	0.2929	0.4186
		Subsample Excluding Financial Holding Firms				
Variables	OLS	Lower quantiles		Median	Upper quantiles	
		10th	25th	50th	75th	90th
ESGRISK	−0.0639 *	−0.0294	−0.0398	−0.0090	−0.0580	−0.1414 *
	(0.0344)	(0.0278)	(0.0295)	(0.0457)	(0.0374)	(0.0811)
SIZE	−0.7088 *	−0.2630	−0.1623	−0.2084	−0.3280	−0.8307
	(0.3606)	(0.1962)	(0.2085)	(0.3227)	(0.2642)	(0.5728)
LEV	0.0537 **	0.0178	0.0198	0.0228	0.0327*	0.0525
	(0.0248)	(0.0140)	(0.0148)	(0.0230)	(0.0188)	(0.0408)
AGE	−0.0093	−0.0142	−0.0147	−0.0180	−0.0260	0.0015
	(0.0192)	(0.0151)	(0.0160)	(0.0248)	(0.0203)	(0.0441)
NPM	0.0725	0.0217 *	0.0171	0.0809 ***	0.1172 ***	0.2244 ***
	(0.0575)	(0.0122)	(0.0129)	(0.0200)	(0.0164)	(0.0355)
OEG	0.0838 *	0.0278 ***	0.0297 ***	0.1675 ***	0.2963 ***	0.3493 ***
	(0.0488)	(0.0074)	(0.0078)	(0.0121)	(0.0099)	(0.0215)
Constant	15.5619 **	5.3315	4.9501	5.6693	9.4994 **	19.3638 *
	(6.2505)	(3.4856)	(3.7045)	(5.7341)	(4.6934)	(10.1768)
Sample size	293	293	293	293	293	293
Adj R-square/Pseudo R^2^	0.2181	0.0764	0.0747	0.1381	0.2997	0.4304

Note: SGR = sustainable growth rate; ESGRISK = ESG Risk Ratings; SIZE = company size; LEV = financial leverage; AGE = firm age; NPM = net profit margin; OEG = growth rate of owner’s equity; Robust (cluster at firm level) standard errors in parentheses; *, ** and *** stands for statistical significance at 10%, 5%, and 1%, respectively.

**Table 4:** The results of inter-quantile regression SGR.

		SGR (Full Samples)	
		Q(90/10)	Q(75/25)
ESGRISK	F-statistics	2.99	1.18
	Sig.	0.0848 *	0.2779
SIZE	F-statistics	2.93	1.80
	Sig.	0.0881 *	0.1803
LEV	F-statistics	0.00	0.58
	Sig.	0.9613	0.4483
AGE	F-statistics	0.02	0.12
	Sig.	0.8807	0.7323
NPM	F-statistics	0.47	0.58
	Sig.	0.4954	0.4461
OEG	F-statistics	34.58	22.80
	Sig.	0.0000 ***	0.0000 ***

Note: SGR = sustainable growth rate; ESGRISK = ESG Risk Ratings; SIZE = company size; LEV = financial leverage; AGE = firm age; NPM = net profit margin; OEG = growth rate of owner’s equity; Q(90/10) = 90th Quant(y)-75th Quant(y); Q(75/25) = 75th Quant(y)-25th Quant(y); * and *** stands for statistical significance at 10%, 5%, and 1%, respectively.

**Table 5:** Regression results of environmentally sensitive and non-sensitive firms’ subsamples.

		Sensitive Firms				
Variables	OLS	Lower Quantiles		Median	Upper Quantiles	
		10th	25th	50th	75th	90th
ESGRISK	−0.1110 *	−0.1573 ***	−0.0681	0.0212	0.0373	−0.1454 **
	(0.0639)	(0.0288)	(0.0618)	(0.0825)	(0.0956)	(0.0579)
SIZE	−0.5819	−0.2176	−0.2261	−0.3722	0.0454	−0.2590
	(0.4098)	(0.2070)	(0.4446)	(0.5934)	(0.6875)	(0.4160)
LEV	−0.0193	−0.0077	0.0065	0.0014	−0.0099	−0.0040
	(0.0380)	(0.0148)	(0.0317)	(0.0423)	(0.0490)	(0.0297)
AGE	0.0726 *	0.0635 ***	0.0485	0.0857	−0.0101	−0.0524
	(0.0410)	(0.0212)	(0.0455)	(0.0608)	(0.0704)	(0.0426)
NPM	0.0305	0.0433 **	0.0224	−0.0136	0.1475 **	0.0921 **
	(0.0418)	(0.0174)	(0.0375)	(0.0500)	(0.0579)	(0.0350)
OEG	0.1001	0.0904 ***	0.0267	0.1041	0.2371 ***	0.3909 ***
	(0.0657)	(0.0226)	(0.0486)	(0.0648)	(0.0751)	(0.0454)
Constant	12.6989 *	4.4351	3.8505	3.4707	0.0736	14.7001 **
	(6.3146)	(3.4387)	(7.3852)	(9.8563)	(11.4186)	(6.9090)
Sample size	42	42	42	42	42	42
Adj R-square/Pseudo R^2^	0.1020	0.226	0.109	0.127	0.265	0.346
		Non-sensitive Firms				
Variables	OLS	Lower quantiles		Median	Upper quantiles	
		10th	25th	50th	75th	90th
ESGRISK	−0.0466	−0.0091	−0.0125	−0.0093	−0.0545	−0.1540
	(0.0334)	(0.0251)	(0.0329)	(0.0496)	(0.0413)	(0.0961)
SIZE	−0.8287 **	−0.2062	−0.1703	−0.3136	−0.5516 **	−1.4146 **
	(0.3602)	(0.1553)	(0.2031)	(0.3068)	(0.2550)	(0.5937)
LEV	0.0418 *	0.0224 *	0.0213	0.0179	0.0252	0.0422
	(0.0242)	(0.0119)	(0.0155)	(0.0234)	(0.0195)	(0.0454)
AGE	0.0055	−0.0162	−0.0123	−0.0074	−0.0102	0.0064
	(0.0204)	(0.0127)	(0.0167)	(0.0252)	(0.0209)	(0.0487)
NPM	0.0605	0.0211 **	0.0166	0.0200	0.0361 **	0.0883 **
	(0.0463)	(0.0098)	(0.0128)	(0.0193)	(0.0161)	(0.0374)
OEG	0.0858 *	0.0318 ***	0.0295 ***	0.1982 ***	0.3292 ***	0.3558 ***
	(0.0508)	(0.0062)	(0.0081)	(0.0123)	(0.0102)	(0.0237)
Constant	17.4497 ***	3.8003	4.4526	7.8576	13.5921 ***	31.7763 ***
	(5.7130)	(2.7007)	(3.5321)	(5.3348)	(4.4350)	(10.3242)
Sample size	281	281	281	281	281	281
Adj R-square/Pseudo R^2^	0.2082	0.0906	0.0668	0.128	0.300	0.422

Note: SGR = sustainable growth rate; ESGRISK = ESG Risk Ratings; SIZE = company size; LEV = financial leverage; AGE = firm age; NPM = net profit margin; OEG = growth rate of owner’s equity; Robust (cluster at firm level) standard errors in parentheses; *, ** and *** stands for statistical significance at 10%, 5%, and 1%, respectively.

**Table 6:** Regression results of large and small firm subsamples.

	SGR	
Variables	Large Firms	Small Firms
ESGRISK	−0.1246 ***	−0.0376
	(0.0464)	(0.0415)
LEV	0.0076	0.0803 **
	(0.0189)	(0.0398)
AGE	0.0061	−0.0452
	(0.0212)	(0.0385)
NPM	0.0220 *	0.3714 **
	(0.0124)	(0.1550)
OEG	6.6679 ***	0.6215
	(1.9030)	(4.4583)
Constant	−0.1246 ***	−0.0376
	(0.0464)	(0.0415)
Sample size	150	173
Adjusted R-squared	0.0118	0.2774
F-value	2.971	3.023
*p*-value	0.0215 **	0.0194 **

Note: SGR = sustainable growth rate; ESGRISK = ESG Risk Ratings; SIZE = company size; LEV = financial leverage; AGE = firm age; NPM = net profit margin; OEG = growth rate of owner’s equity; Robust (cluster at firm level) standard errors in parentheses; *, ** and *** stands for statistical significance at 10%, 5%, and 1%, respectively.

## Data Availability

Data sharing not applicable.
